# Long Non-Coding RNA MEG3 in Cellular Stemness

**DOI:** 10.3390/ijms22105348

**Published:** 2021-05-19

**Authors:** Pei-Fang Hsieh, Cheng-Chia Yu, Pei-Ming Chu, Pei-Ling Hsieh

**Affiliations:** 1 Department of Urology, E-Da Hospital, Kaohsiung 82445, Taiwan; n52022@gmail.com; 2 Department of Medical Laboratory Science and Biotechnology, Chung-Hwa University of Medical Technology, Tainan 71703, Taiwan; 3 Institute of Oral Sciences, Chung Shan Medical University, Taichung 40201, Taiwan; ccyu@csmu.edu.tw; 4 Department of Dentistry, Chung Shan Medical University Hospital, Taichung 40201, Taiwan; 5 School of Dentistry, Chung Shan Medical University, Taichung 40201, Taiwan; 6 Department of Anatomy, School of Medicine, China Medical University, Taichung 404333, Taiwan; pmchu@mail.cmu.edu.tw

**Keywords:** long non-coding RNA MEG3, embryonic stem cells, mesenchymal stem cells, cancer stem cells

## Abstract

Long non-coding RNAs (lncRNAs) regulate a diverse array of cellular processes at the transcriptional, post-transcriptional, translational, and post-translational levels. Accumulating evidence suggests that lncRNA MEG3 exerts a large repertoire of regulatory functions in cellular stemness. This review focuses on the molecular mechanisms by which lncRNA MEG3 functions as a signal, scaffold, guide, and decoy for multi-lineage differentiation and even cancer progression. The role of MEG3 in various types of stem cells and cancer stem cells is discussed. Here, we provide an overview of the functional versatility of lncRNA MEG3 in modulating pluripotency, differentiation, and cancer stemness.

## 1. Introduction

Maternally expressed gene 3 (MEG3), also known as gene trap locus 2 (Gtl2), was identified by gene trap insertion, and tightly linked to paternally expressed Delta-like 1 (Dlk1) gene at chromosome 14q32.3 within Dlk1-Dio3 locus [1,2,3]. The Dlk1 gene encodes a transmembrane protein that contains multiple epidermal growth factor repeats and belongs to the Notch signaling family [4]. On the other hand, MEG3 is widely expressed during development in the paraxial mesoderm, the developing central nervous system, and the epithelia of the salivary glands, kidney, and pancreas [1]. Due to the lack of known functional proteins encoded by the small open reading frames of this gene transcript, MEG3 has been considered as a long non-coding RNA (lncRNA) for tumor suppression [5].

**Figure 1:**
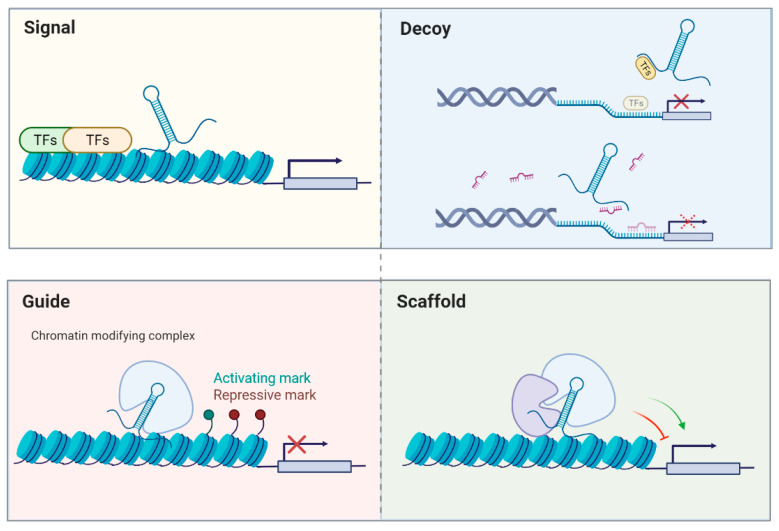
General modes of action of lncRNAs. (Adapted from [7]).

Conventionally, non-coding RNAs can be divided into lncRNAs that have more than 200 nucleotides in length and the remaining RNAs are recognized as “small” non-coding RNAs, such as microRNAs (miRNAs; ~22 nucleotides), piwiRNAs (piRNAs; ~26–31 nucleotides), or small nucleolar RNAs (snoRNAs; ~60–300 nucleotides). MiRNAs have been known to repress the expression of a target gene with partially complementary binding sites in its 3′ untranslated region (3′UTR) [6]. As for lncRNAs, diverse functions have been discovered to regulate various biological processes, including serving as scaffolds, molecular signals, guides, or decoys (see review [7]; Figure 1). Briefly, some lncRNAs occur at a certain time and space to integrate diverse stimuli (signal), or they may initiate cis- or trans-regulation of gene expression (guide). Additionally, they can function as platforms to assemble relevant molecular components to promote or repress gene expression (scaffold). Moreover, lncRNAs may bind to their targets or miRNAs (decoy) to inhibit their action. A similar concept proposes that lncRNAs may be one kind of competing endogenous RNA (ceRNAs) and serve as miRNA sponges to modulate the distribution of miRNAs on their targets via miRNA response elements [8]. The interplay between lncRNAs and miRNAs has been extensively studied and many reports indicate that lncRNAs may modulate the expression of certain mRNAs by acting as molecular decoys and sequestering miRNAs to affect various disease states.

A number of studies have suggested that the expression of MEG3 is downregulated in several types of cancer cells [5,9,10], and DNA methylation has been proven to play a significant role in silencing MEG3 in tumors [11]. Aside from functioning as a tumor suppressor, emerging evidence suggests that MEG3 may be involved in the regulation of stemness. In this review, we briefly summarized recent studies about the effect of MEG3 on the differentiation of embryonic and mesenchymal stem cells as well as the aggressiveness of cancer stem cells. We focused on targets/interacting factors and molecular mechanisms to provide an insight into the multiple functions of MEG3 in cellular stemness.

## 2. Stem Cells

### 2.1. Embryonic Stem Cells

During embryonic development and adulthood, MEG3 has been detected in the mouse forebrain [12] and corticospinal neurons [13], indicating MEG3 may play a part in the maturation of the central nervous system. Besides, MEG3 has been found to be highly expressed in human embryonic stem cells (hESCs) and to affect their neural differentiation capacities [14,15]. Embryoid bodies derived from hESCs with undetectable MEG3 have been found to exhibit unusual morphologies along with decreased expression of the neural stem cell marker, paired box 6 (PAX6) [14]. Knockdown of MEG3 in hESCs results in the downregulation of various neural lineage genes. Additionally, the neural lineage-like cells derived from hESCs with undetectable MEG3 display a lower expression of lineage-specific markers and form fewer neurites [14], suggesting the significance of MEG3 in the neural lineage differentiation of hESCs.

Additionally, MEG3 has been found to interact with polycomb repressive complex-2 (PRC2) via jumonji, AT rich interactive domain 2 (JARID2), and facilitate the recruitment of PRC2 on chromatin [15,16]. The potential site where MEG3 interacts with PRC2 has also been described [17]. JARID2 is a crucial modulator of PRC2 enzymatic activity and is required for the multi-lineage differentiation of ESCs [18]. On the other hand, PRC2 is a histone methyltransferase for epigenetic silencing that works by providing the H3K27me3 repressive epigenetic mark [19]. A large number of lncRNAs have been thought to facilitate the recruitment of PRC2 to the correct chromatin sites [20], and MEG3 is one of them. It serves as a scaffold and bridges the PRC2/JARID2 complex to sustain the rostral motor neuron cell fate through silencing the epigenetic state of progenitor genes [16]. Besides this, the lack of MEG3 in human induced pluripotent stem (iPS) cells has been demonstrated to alter the chromatin distribution of JARID2, PRC2, and H3K27me3 [15]. These findings indicate that MEG3 is essential for the transition from stem cell pluripotency to differentiation, by mediating the proper recruitment and assembly of PRC2 on target genes.

### 2.2. Hematopoietic Stem Cells

MEG3 is reported to be highly expressed in hematopoietic stem cells (HSCs) and gradually repressed in early progenitors [21]. Qian and colleagues demonstrated that the Dlk1-Gtl2 locus is predominantly enriched in both fetal HSCs and adult long-term repopulating-HSCs. Additionally, loss of the Dlk1-Gtl2 locus results in deficiency and impaired long-term reconstitution capacity in fetal liver HSCs. Their results reveal that the Dlk1-Gtl2 locus seems to be crucial in maintaining the functionality of long-term HSCs by suppressing the phosphatidylinositol 3-kinase (PI3K)/AKT/mammalian target of the rapamycin (mTOR) signaling and subsequently restricting mitochondrial biogenesis, which prevents excessive reactive oxygen species generation [22]. Of note, silencing MEG3 expression does not compromise adult hematopoiesis after the establishment of adult homeostatic hematopoiesis in an inducible MxCre Meg3^mat-flox/pat-wt^ mouse model to induce deletion of MEG3 [21]. Sommerkamp et al. suggest that MEG3 is dispensable for the embryonic establishment of hematopoiesis after the endothelial cell-to-HSC transition [21].

### 2.3. Bone Marrow-Derived Mesenchymal Stem Cells

A growing body of evidence suggests that MEG3 may be critical to the osteogenesis of bone marrow mesenchymal stem cells (BMSCs), but its role has not been fully elucidated, as it seems to positively and negatively modulate this process. The expression of MEG3 has been known to be associated with various osteogenic markers, such as runt-related transcription factor 2 (RUNX2), osterix (Osx), and osteocalcin (OCN) [23,24]. Numerous studies have investigated the possible mechanisms underlying changes in MEG3 during osteogenic differentiation. It has been shown that DEP domain-containing mTOR interacting protein (DEPTOR) binds to a specific region (−1000 bp–0) of the MEG3 promoter to inhibit its transcription during osteogenesis of BMSCs. As such, DEPTOR can impede the MEG3-mediated activation of bone morphogenic protein 4 (BMP4) signaling, which inhibits osteogenic differentiation [25]. Another study shows that DNA cytosine-5-methyltransferases 1 (DNMT1) mediates the hypermethylation of MEG3 promoter in BMSCs, leading to downregulation of MEG3 and BMP4 [26]. In respect to the mechanism of MEG3-regulated osteogenesis, Zhuang et al. revealed that MEG3 acts as a cofactor to affect the activity of sex-determining region Y (SRY)-box 2 (SOX2) and to disrupt the binding of SOX2 on the BMP4 promoter, which enhances BMP4 expression and promotes osteogenic differentiation [24]. MEG3 also holds the potential to inhibit osteogenesis by directly targeting miR-133a-3p and diminishing the solute carrier family 39 member 1 (SLC39A1) [23], which encodes Zinc transporter ZIP1, an inducer that initiates an osteogenic lineage from MSCs [27].

Moreover, MEG3 modulates chondrocyte differentiation by sequestering miRNAs. It has been demonstrated that the expression of MEG3 is gradually elevated when BMSCs differentiate into chondrocytes, while miR-129-5p is downregulated [28]. Work by Zhu et al. showed that forced expression of MEG3 elevates the expressions of various chondrocyte-related genes, and one of them is RUNX1, a target gene of miR-129-5p [28]. Apart from regulating the miR-129-5p/RUNX1 axis to promote chondrocyte differentiation, MEG3 also participates in endothelial differentiation. It has been shown that the expression of MEG3 is decreased during the transition from BMSCs to endothelial cells [29]. In a rat model of diabetic erectile dysfunction, the expression of MEG3 was found to decrease in the corpus cavernosum tissues of rats receiving intracavernous implantation of BMSCs [29]. MEG3 is able to interact with forkhead box protein M1 (FOXM1) and accelerate the degradation of FOXM1 via ubiquitination. The reduced expression of MEG3 leads to upregulation of FOXM1, which physically interacts with the vascular endothelial growth factor (VEGF) promoter and transcriptionally activates its expression [29]. Taken together, MEG3 mediates multi-lineage differentiation by serving as a cofactor or a miRNAs sponge to manage target expression.

### 2.4. Adipose-Derived Mesenchymal Stem Cells

A progressive increase in MEG3 expression during the replicative senescence of human adipose-derived mesenchymal stem cells (hADSCs) has been observed previously, and histone deacetylation is implicated in the alteration of MEG3, rather than the methylation levels in the differentially methylated region (DMR) domains [30]. MEG3 also elicits apoptosis of hADSCs by upregulating p53, which, in turn, affects the downstream apoptotic Bcl-2/Bax pathway [31]. Similarly, the silencing of MEG3 markedly decreases the H_2_O_2_-induced apoptosis by regulating p53 [31], indicating MEG3′s imperative role in hADSCs’ survival under oxidative stress. The expression of MEG3 not only harmonizes the cellular senescence/apoptosis of hADSCs, but is also involved in the decision of adipogenic and osteogenic lineage differentiation of hADSCs. It has been shown that MEG3 inhibits adipogenesis and promotes osteogenesis via directly binding to miR-140-5p [32], which may enhance BMP2-mediated osteogenesis [33]. Li et al. also demonstrated that new bone formation was greatly reduced in the nude mice receiving hADSCs transfected with sh-MEG3, compared to the control group after 8 weeks of transplantation, which was in line with the in vitro finding [32].

### 2.5. Dental Tissue-Derived Stem Cells

Aside from on hematopoietic, bone marrow- or adipose-derived mesenchymal stem cells, MEG3 also exerts regulatory effects on dental tissue-derived stem cells, such as dental pulp, dental follicle, and periodontal ligament stem cells. Deng et al. showed that MEG3 interacts with the enhancer of zeste homolog 2 (EZH2), a functional enzymatic component of PRC, and plays an inhibitory role in the osteogenesis of human dental follicle stem cells (hDFSCs), as EZH2 inhibits β-catenin and Wnt ligands expression via H3K27me3 deposition [34]. Another study demonstrated that MEG3 increases the Smad ubiquitin regulatory factor 1 (SMURF1) expression, an inhibitor of osteoblast differentiation [35], by serving as a ceRNA to sequester miR-543 in human dental pulp stem cells (hDPSCs) [36]. Regarding the periodontal ligament stem cells (PDLSCs), MEG3 is known to hinder osteogenic differentiation by interacting with heterogeneous nuclear ribonucleoprotein I (hnRNPs), which results in the downregulation of BMPs [37]. Liu et al. showed that hnRNPI stabilizes BMP2 mRNA and increases its protein expression by a post-transcriptional regulation mechanism. They demonstrated that MEG3 competes with BMP2 mRNA for hnRNPI, leading to repression of BMP2 [37]. As mentioned above, MEG3 coordinates osteogenic differentiation in stem cells by targeting multiple molecules. It has been demonstrated that overexpression of MEG3 promotes the osteogenesis of PDLSC via sponging miR-27a-3p for the upregulation of insulin-like growth factor 1 (IGF1) and activation of PI3K/Akt signaling [38].

### 2.6. Synovium-Derived Mesenchymal Stem Cells

After successfully isolating the multipotent MSCs from the synovium of knee joints [39], synovium-derived mesenchymal stem cells (SMSCs) have been considered as a promising type of stem cells for cartilage regeneration [40]. It has been demonstrated that MEG3 is able to repress the chondrogenic differentiation of SMSCs by directly interacting with EZH2 and epigenetically suppressing tribbles pseudokinase 2 (TRIB2) via EZH2-mediated H3K27me3 [41]. Moreover, knockdown of MEG3 or forced expression of TRIB2 induces the chondrogenic differentiation of SMSCs and elevates the expression of two major components in the articular cartilage: collagen type II α 1 chain (Col2A1), and aggrecan [41]. This finding suggests that modulation of MEG3 may be a viable method to enhance chondrogenesis. The aforementioned stem cells and the associated mechanism are listed in Table 1.

## 3. Cancer Stem Cells

### 3.1. Introduction

The cancer stem cell (CSC) theory was proposed after the discovery of CSCs in various types of cancer, such as leukemia [42,43] and breast cancer [44]. CSC theory posits that tumor growth is driven by a small number of cells in tumor tissues, as they have the ability to self-renew and differentiate into other types of cells that comprise the tumor. It has been thought that cancer recurrence and drug resistance are attributed to the existence of CSCs. Furthermore, mounting evidence indicates that there is a strong connection between CSCs and epithelial-to-mesenchymal transition (EMT), which is associated with metastasis by initiating cell migration and invasion. It has been shown that overexpression of various EMT transcription factors, such as Slug and Snail, contributes to the stemness in cancer cells [45,46]. Although there is a lack of clear evidence demonstrating the effect of MEG3 on cancer stemness, several studies have revealed that modulation of MEG3 affects the expression of EMT transcription factors and mesenchymal properties. For instance, it has been shown that ectopic expression of MEG3 both decreases the expression of Snail and increases the expression of E-cadherin in esophageal squamous cell carcinoma cells [47]. Similarly, the overexpression of MEG3 downregulates the expression of N-cadherin, Vimentin, and Snail, while silencing MEG3 increases the expression of these markers in glioma cells [48] and cholangiocarcinoma cells [49]. Additionally, forced expression of MEG3 has been shown to decrease the sphere-forming ability and the expression of CSCs’ markers, such as Nanog and Oct4, as well as being shown to enhance the chemosensitivity in pancreatic cancer cells [50]. Collectively, these studies imply that downregulation of MEG3 in cancer cells may convert the non-CSCs into a CSC phenotype.

### 3.2. Liver Cancer Stem Cells

Recent studies have demonstrated that MEG3 inhibits the growth in liver CSCs via regulation of telomerase activity. Jiang et al. showed that MEG3 increases the binding ability of RNA polymerase II to the p53 promoter region, which elevates the expression of p53 in liver CSCs [51]. Subsequently, MEG3 increases the H3K27me modification in the telomerase reverse transcriptase (TERT) promoter region through p53, thereby inhibiting the expression of TERT. Moreover, MEG3 reduces the binding of telomerase RNA component (TERC) to TERT and inhibits the activity of telomerase [51]. They also revealed that MEG3 can form circular RNA. In another study by the same group, they showed that circMEG3 downregulates the expression of the telomerase component Cbf5 in human liver CSCs, thereby restricting the lifespan of telomeres in liver CSCs [52]. Furthermore, they showed that the expression levels of key pluripotency factors, such as Oct4, SOX2, and Nanog, are markedly reduced in the liver CSCs with an increased CircMEG3 [52]. Their work demonstrates that MEG3 regulates the cancer stemness in liver cancer via the suppression of telomerase activity.

### 3.3. Head and Neck Cancer Stem Cells

Current evidence indicates that MEG3 serves as a tumor suppressor to regulate the cancer stemness in head and neck cancers. It has been reported that the expression of MEG3 is downregulated in the tissues and stemlike cells (GSCs) of glioblastoma [53]. Buccarelli et al. demonstrated that MEG3 restoration mitigates the tumorigenic abilities of GSCs. Additionally, they showed MEG3 induces a marked downregulation of EMT marker, vimentin, and stemness markers Sox2 and Notch, along with an increase in β-actin in GSCs [53]. In oral cancer stem cells (OCSCs), it has been shown that the expression of MEG3 is reduced in OCSCs, which is associated with poor prognosis [54]. Furthermore, MEG3 directly interacts with miR-421 to affect several CSCs features, such as self-renewal or invasion capacities [54]. MiR-421 has been shown to participate in the decision of ESCs’ fate through direct repression of Oct 4 and BMP signaling [55]. As such, MEG3 may regulate the fate of CSCs by modulation of Oct4 or BMP pathway via miR-421.

### 3.4. Ovarian Cancer Stem Cells

In ovarian cancer stem cells, MEG3 also interacts with miR-421 [56]. It appears that there is a reciprocal regulation of miR-421 and MEG3, as the forced expression of MEG3 inhibits the level of miR-421 in OCSCs [54], and overexpression of miR-421 results in a reduction in MEG3 and platelet-derived growth factor receptor A (PDGFRA) in ovarian cancer stem cells [56]. PDGF signaling has been known to control stemness, metastatic potential, and chemoresistance in ovarian cancer stem cells [57], so upregulation of miR-421 may inhibit cancer stemness by inhibiting PDGF signaling. It is worthy of note that the expression of MEG3 is overexpressed in the tumor samples of ovarian cancer, and downregulated in the anisomycin-treated ovarian cancer stem cells [56]. As such, whether MEG3 functions as a tumor suppressor in ovarian cancer remains to be determined.

### 3.5. Lung Cancer Stem Cells

The expression of MEG3 is decreased in lung cancer stem cells (LCSCs) [58]. Zhao et al. showed that knockdown of MEG3 elevates the expression levels of stemness marker Oct4, and CD133 [58]. Moreover, upregulation of MEG3 inhibits the migration and invasion of LCSCs through upregulation of SLC34A2 expression, by sponging miR-650 [58]. SLC34A2 possesses an inhibitory effect on cell growth, motility, and invasiveness of lung cancer cells, which may be due to the regulation of PI3K/Akt/mTOR or Ras/Raf/MEK/Erk signal pathways [59]. The aforementioned CSCs and the MEG3-mediated molecular mechanism are listed in Table 2.

## 4. Conclusions

**Figure 2:**
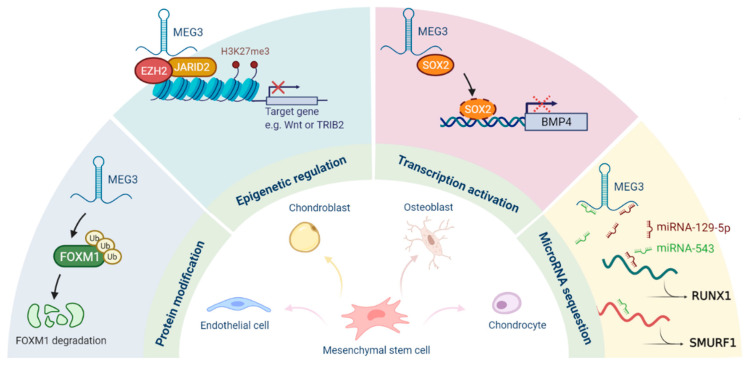
Roles of MEG3 in the regulation of stemness.

Multiple mechanisms for the regulation of cellular stemness by MEG3 have been gradually discovered (Figure 2). Firstly, MEG3 acts as a scaffold to bridge the PRC2/JARID2 complex and to facilitate the recruitment of PRC2 to the target sites for epigenetic silencing. Secondly, it functions as a cofactor to hinder the binding of SOX2 on the BMP4 promoter and to promote osteogenic differentiation. MEG3 also serves as a ceRNA to regulate the expression of target genes by sequestering various microRNAs, such as miR-129-5p, miR-140-5p, miR-543, and miR-421. It can also interact with FOXM1 to induce the degradation of FOXM1 via ubiquitination. As for MEG3-mediated cancer stemness, p53 and miR-421 may contribute to its regulatory capacity. Several studies have shown that the expression of MEG3 is repressed in CSCs or cancer tissues, indicating that MEG3 may have inhibitory effects on cancer stemness, at least in the liver, head and neck, and lung cancers.

## Figures and Tables

**Table 1:** The effect of MEG3 on the differentiation of various types of stem cells.

Author, Year	Cell Types	Differentiation	Change during Differentiation	Interacting Factor/Upstream Regulator	Molecular Mechanisms	Expression in Disease
Li, 2017 [32]	hADSCs	Adipogenic	Downregulated	miR-140-5p		
		Osteogenic	Upregulated			
Zhuang, 2015 [24]	hBMSCs	Osteogenic	Upregulated	Sox2	MEG3 upregulates BMP4 transcription by disrupting SOX2 on the BMP4 promoter	Downregulated in BMSCs from patients with multiple myeloma
Wang, 2017 [23]	hBMSCs	Osteogenic	Downregulated	miR-133a-3p	MEG3 increases the expression of miR-133a-3p by direct binding, which may downregulate SLC39A1	Upregulated in BMSCs from patients with postmenopausal osteoporosis
Chen, 2018 [25]	hBMSCs	Osteogenic		DEPTOR	DEPTOR inhibits osteogenic differentiation by directly binding to the MEG3 promoter and suppressing the MEG3-mediated activation of BMP4 signaling	
Li, 2021 [26]	hBMSCs	Osteogenic		DNMT-1	DNMT1 mediates the hypermethylation and downregulation of MEG3 to inhibit osteogenic differentiation via reducing BMP4	Downregulated in BMSCs from patients with pediatric aplastic anemia
Zhu, 2021 [28]	hBMSCs	Chondrogenic	Upregulated	miR-129-5p	MEG3 modulates the miR-129-5p/RUNX1 axis to induce the differentiation of BMSCs into chondrocytes	
Sun, 2018 [29]	Rat BMSCs	Endothelial	Downregulated	FOXM1	MEG3 increases the ubiquitination of FOXM1 protein and decreases FOXM1 protein level	Downregulated in the corpus cavernosum tissues using the diabetic rat model of erectile dysfunction
Deng, 2018 [34]	hDFSCs	Osteogenic		EZH2/ Wnt	MEG3 interacts with EZH2 to suppress β-catenin expression by increasing H3K27me3 at the Wnt promoter	
Zhao, 2020 [36]	hDPSCs	Osteogenic	Downregulated	miR-543	MEG3 sequesters miR-543 to upregulate the expression of SMURF1	
Liu, 2019 [37]	hPDLSCs	Osteogenic (Mineralizing solution)	Downregulated	hnRNPI	MEG3 competes with BMP2 for hnRNPI, which leads to downregulation of BMP2 (hnRNPI can stabilize BMP2)	
Liu, 2019 [38]	hPDLSCs	Osteogenic (StemProTM Osteogenesis Differentiation Kit)	Upregulated	miR-27a-3p	MEG3 sequesters miR-27a-3p to upregulate the expression of IGF-1 and activate PI3K/Akt signaling	Downregulated in periodontitis periodontal tissues
You, 2019 [41]	hSMSCs	Chondrogenic	Downregulated	EZH2/ TRIB2	MEG3 interacts with EZH2 to inhibit TRIB2 expression by increasing H3K27me3 at the TRIB2 promoter	

**Table 2:** The role of MEG3 in various types of cancer stem cells.

Author, Year	Cell Types	Expression	Role in Cancer	Targets/Interacting Factors	Molecular Mechanisms
Jiang, 2020 [51]	Liver CSCs		Tumor suppressor	p53	MEG3 increases the methylation modification of histone H3 at the 27th lysine via p53, which leads to downregulation of telomerase activity
Jiang, 2020 [52]	Liver CSCs	CircMEG3 expression is downregulated	Tumor suppressor	METTL3	CircMEG3 inhibits the expression of m6A methyltransferase METTL3 dependent on HULC, leading to suppression of Cbf5 and reduction of telomerase activity and telomere lifespan
Buccarelli, 2020 [53]	Glioblastoma CSCs	Downregulated in the tissues of glioblastoma and CSCs	Tumor suppressor		MEG3 may contribute to the reduction of stemness factors, such as Sox2 and Notch, as well as EMT factors, such as vimentin
Chen, 2021 [54]	Oral CSCs	Downregulated in the tissues of oral cancer	Tumor suppressor	miR-421	MEG3 impedes the action of miR-421
Zhao, 2019 [58]	Lung CSCs	Downregulated in lung CSCs	Tumor suppressor	miR-650	MEG3 positively regulates the expression of SLC34A2 by sponging miR-650
